# Brain morphological characteristics predicting clinical response to selective serotonin reuptake inhibitors or cholinesterase inhibitors: A study of electronic medical records in patients with cognitive disorders

**DOI:** 10.1016/j.inpsyc.2025.100105

**Published:** 2025-06-25

**Authors:** Kengo Onda, Jill S. Chotiyanonta, Hannah P. Cowley, Yuto Uchida, Milap A. Nowrangi, Roy Adams, Constantine G. Lyketsos, Peter P. Zandi, Kenichi Oishi

**Affiliations:** a Department of Radiology, Johns Hopkins University School of Medicine, Baltimore, MD, USA; b Research and Exploratory Development Department, Johns Hopkins University Applied Physics Laboratory, Laurel, MD, USA; c Department of Psychiatry and Behavioral Sciences, Johns Hopkins University School of Medicine, Baltimore, MD, USA; d Richman Family Precision Medicine Center of Excellence in Alzheimer's Disease, Johns Hopkins University School of Medicine, Baltimore, MD, USA; e Department of Neurology, Johns Hopkins University School of Medicine, Baltimore, MD, USA

**Keywords:** Alzheimer's disease, Cholinesterase inhibitor, Electronic health record, Magnetic resonance imaging, Psychiatric symptom, Treatment response, Selective serotonin reuptake inhibitor, Vascular dementia

## Abstract

**Objectives:**

Current treatments for cognitive and neuropsychiatric symptoms in Alzheimer's disease and related dementias (ADRD), such as cholinesterase inhibitors (CEIs) and selective serotonin reuptake inhibitors (SSRIs), show inconsistent effectiveness, necessitating a personalized therapy approach. We aimed to develop predictive models using MRI-derived brain neuroanatomical features and clinical data to forecast responses to CEIs and SSRIs in ADRD patients.

**Design and setting:**

This was a retrospective observational analysis of electronic health records (EHRs) and MRI data conducted within Johns Hopkins Medical Systems.

**Participants:**

Cohort 1 comprised 179 patients prescribed CEIs or SSRIs for the first time with over a year of follow-up. Cohort 2 included 1244 patients with similar criteria to explore clinical characteristics linked to MRI features affecting treatment benefits.

**Measures:**

Medication efficacy was assessed via a Likert scale based on EMR descriptions. We quantified brain volumes across 280 anatomical areas on T1-weighted MRIs and applied an elastic net model after harmonizing volumes with the ComBat model. Predictive model efficacy was evaluated using receiver operating characteristic (ROC) analysis.

**Results:**

Preserved volume in the nucleus basalis of Meynert correlated with better responses to both CEIs and SSRIs. Specific white matter volumes related to CEI benefits, while SSRI efficacy was linked to gray matter in the Nucleus Accumbens and frontal cortex. Area under ROC curve values were 0.82 for CEI and 0.75 for SSRI predictions. Older age and vascular factors were associated with reduced medication benefits.

**Conclusions:**

MRI-derived neuroanatomical features effectively predict medication responses in ADRD, potentially enabling more tailored treatment strategies.

## Introduction

Alzheimer's disease (AD) and related dementias (ADRD), including vascular dementia (VaD) and mixed dementia, progressively impair cognitive abilities, leading to disability and loss of independence [1-6]. While new treatments, such as anti-amyloid immunotherapies [7], are emerging, their effectiveness remains debated. Current treatments focus on neurotransmission, with cholinesterase inhibitors (CEIs) enhancing cognitive function through cholinergic neurotransmission [8,9] and selective serotonin reuptake inhibitors (SSRIs) alleviating psychiatric symptoms by facilitating serotoninergic neurotransmission [10,11]. However, their efficacy varies [12,13], with many patients experiencing adverse effects [14-17]. Predicting patient subgroups that respond favorably to these treatments is crucial for maximizing benefits and minimizing adverse effects, a strategy known as precision medicine [18].

Several markers, including clinical (e.g., symptom severity, blood pressure, pupillary dilation, age) and genetic (APOE, BDNF, or CYP450) features, have been proposed as predictors of treatment response but need further validation [13]. Quantifying brain neuroanatomical features through magnetic resonance imaging (MRI) has succeeded in identifying subgroups of AD related to clinical, cognitive, and neuropsychiatric characteristics [19-28]. However, little is known about the capability of brain phenotyping to predict responses to pharmacological interventions, with a limited number of studies reporting mixed results. While some research indicates that patients with milder cognitive symptoms and less atrophy in limbic and paralimbic structures are more likely to respond to CEI [29-31], others suggest a link between altered cholinergic systems in the basal forebrain and positive CEI response [32,33]. These findings underscore the need to study brain anatomy in larger, more representative data sets utilizing real-world electronic health records (EHR), since such a study design provides a valuable opportunity to explore the critical questions that are generally underpowered and unable to be answered in a prospective research setting [34].

This study aims to develop predictive models for responses to CEIs and SSRIs using brain MRIs, while also examining associated clinical characteristics. We retrospectively analyzed EHRs from patients who started on these treatments, assessing the relationship between drug efficacy [35] and regional brain volume. Brain volumes were measured using a multi-atlas label fusion method [36], and an elastic net model identified regions linked to treatment benefits. The models were then applied to a larger cohort to explore correlations between predicted efficacy and clinical features, including demographics and vascular risk factors.

## Materials and methods

This study utilized EHRs from patients of the Johns Hopkins Health System, with ethical approval obtained from the Institutional Review Board. Due to the retrospective nature of the data collected during routine clinical care, a waiver of consent was granted to ensure privacy.

The study included two cohorts. Cohort 1 consisted of a small group used to create and validate models for predicting the efficacy of CEI or SSRI treatment. Due to the limited number of participants, analyzing differences in clinical characteristics was challenging. In contrast, Cohort 2 included over 1000 participants, allowing the application of prediction models and identification of clinical characteristics of those likely to benefit from CEI or SSRI treatment. Further details on both cohorts are provided in the following sections.

### Cohort 1 for predictive modeling

This cohort was selected from a previously reported [35] cohort of patients with cognitive impairments who were prescribed CEI or SSRI for the first time between 2013 and 2021. To generate this original cohort, we used the Richman Family Precision Medicine Center of Excellence in Alzheimer's Disease (PMCoE) database via the Precision Medicine Analytics Platform (https://ictr.johnshopkins.edu/service/informatics/pmap/), which archives EHRs from the Johns Hopkins Health System as a Health Insurance Portability and Accountability Act (HIPPA)- defined limited dataset.

Patients were included if they (1) had a recorded prescription of CEI or SSRI when they were at least age 50 (index date), (2) had any encounter diagnosis of MCI or dementia (ICD-10 codes G30, G31, or F01–03) within a year before and after index date, (3) underwent a T1-weighted brain MRI scan within one year before or after the index date, and (4) had at least one medical encounter within a year before and after the index date. Patients were excluded if (1) their MRI scans had severe motion artifacts, (2) their MRI scans had a slice thickness of 5 mm or greater, or (3) they had a prescription of a serotonin-noradrenaline reuptake inhibitor (SNRI) in the three months prior to the index date. For further details of the cohort, see [35].

### Cohort 2 to explore clinical characteristics associated with brain MRI features related to the benefits of CEI or SSRI

The imaging models to forecast CEI or SSRI response, developed from Cohort 1, were applied to T1-weighted MRIs of Cohort 2 to calculate their CEI and SSRI scores, indicative of favorable responses to CEI and SSRI. Cohort 2 was obtained from the PMCoE database with the following inclusion criteria: (1) evaluated with a T1-weighted MRI scan at least once, (2) seen in the Johns Hopkins Bayview Memory and Alzheimer's Treatment Center (MATC), or (3) seen in a Johns Hopkins Community Physicians (JHCP) clinic with a cognitive disorder diagnosis, or (4) seen in a JHCP clinic and over the age of 65 years and therefore at significantly increased risk of a cognitive disorder. The patients included in Cohort 1 were excluded from Cohort 2. Since vascular risk factors and vascular diseases are known to be associated with cognitive dysfunction [37,38], we included them as clinical characteristics of interest. Please note that participants in Cohort 2 were included without consideration of whether they were prescribed CEIs or SSRIs.

### Clinical benefits of treatment with CEI or SSRI (Cohort 1)

The clinical note at the follow-up visit within one year after the index date was used to evaluate the benefits of medications. If there were multiple visits that met this criterion, the visit closest to 24 months after the index date was chosen. From clinical notes in free text format, keywords related to cognitive function changes, psychiatric conditions, or both were extracted and reviewed following the procedure elaborated in [35] by a research data analyst (JSC). We employed a 3-point Likert scale, named the NOte-based evaluation method for Treatment Efficacy (NOTE) [35], categorizing responses as 1 =improved, 2 =no change, and 3 =worse, to convert clinical note data into a simple, quantifiable measure of medication response. Given the cognitive decline of dementia, CEI-benefitted individuals were those whose cognitive symptoms either improved or did not change. CEI non-benefitted individuals were those whose cognitive symptoms worsened. Patients benefitting from SSRIs (SSRI-benefitted) were identified based on improved psychiatric symptoms. SSRIs non-benefitted patients' psychiatric symptoms showed no change or worsened.

### MRI variables (Cohorts 1 and 2)

T1-weighted images on clinical MRI scanners from the PMCoE database were collected. The field strength, vendors, and scan parameters, including resolution, repetition time, echo time, and inversion time, are summarized in Table 2. The whole brain MRI was parcellated into 280 regions using MRICloud platform (https://mricloud.org/) [39], which warps multiple atlases into linearly arranged images using Large Deformation Diffeomorphic Metric Mapping [40] followed by a multi-atlas fusion algorithm [36]. Measured volumes were normalized by the total brain volume. OPNested Combat [41] was applied to the normalized regional volume to account for potential bias effects related to differences in the field strength (1.5 T and 3 T), MRI scanner vendors (Siemens, Philips, and GE), and slice thickness (≦1.2 mm, 1.3 - 2.4 mm, and ≧ 2.5 mm). Post-Combat application, regional volumes were converted into z-scores, utilizing the mean and standard deviation (SD) from Cohort 1.

### Demographic and clinical covariates (Cohorts 1 and 2)

We summarized two cohorts using demographic and clinical variables related to dementia development and measurement. Demographics included age and sex from intake forms. To measure vascular risk, we include systolic and diastolic blood pressure, body mass index (BMI), HbA1C, LDL, and indicators for hypertension, obesity, diabetes, and dyslipidemia diagnoses. Additionally, we included indicators for diagnoses of chronic kidney disease (CKD), peripheral vascular disease (PVD), heart failure, Alzheimer’s disease (ICD-10: G30), vascular dementia (ICD-10: F01), mixed dementia (diagnsoses for more than one type), and “other” dementia, which includes all non-Azheimer’s and non-vascular dementias (including unspecified). For all lab values and vitals, the most recent value taken within the prior year were used. Diagnosis indicators were positive if the patient had at least one relevant encounter diagnosis or hospital billing code in one year prior to the index date. The ICD-10 codes for hypertension, obesity, diabetes, dyslipidemia, CKD, PVD, and heart failure were pulled from the Chronic Conditions Data Warehouse list of chronic and disabling conditions (https://www2.ccwdata.org/web/guest/condition-categories).

### Statistical analyses

For descriptive statistics, continuous variables were expressed as mean ± SD or median (interquartile range) based on the normality of data distribution. Demographics and clinical characteristics were compared among the CEI and SSRI (Cohort 1) and Cohort 2, using the Kruskal-Wallis or Fisher's exact tests with post-hoc Bonferroni correction for multiple comparisons. Significance was set at a Bonferroni-corrected p-value of < 0.05.

For Cohort 1, permutation tests were performed to identify regions with significant differences in the z-score-converted brain volumes between the benefitted and non-benefitted groups. P-values were calculated based on 100,000 permutations. The significance level was set at p-values of < 0.05. Heatmaps were created to visualize the difference in their z-score-converted volumes between benefitted and non-benefitted groups. Heatmaps color-coded by p-values in these brain anatomical structures were also visualized.

Elastic net regularized regression was utilized to construct two models on Cohort 1: one for predicting the benefits of CEI and another for predicting the benefits of SSRI. The ElasticNetCV class in the Python scikit-learn library was used for the modeling. To train the models, z-score-converted volumes of 280 anatomical regions were employed as independent variables, and response to medications (benefitted or non-benefitted) was used as a dependent variable. The CEI and SSRI prediction models provide scores representing the likelihood of a positive response: CEI score and SSRI score, respectively. To estimate the model performance, the stratified five-fold cross-validation was used to calculate the area under the curve (AUC) from the receiver operating characteristic (ROC) analysis. The optimal cut-off value was determined based on the Youden index from the mean ROC curve. We used DeLong’s test to assess the statistical significance of the AUC compared to random classification.

We applied the trained elastic net regression models to the data from Cohort 2 MRI to examine the relationship between the CEI and SSRI scores, as well as the association of these scores with clinical characteristics. For continuous variables, we used Pearson’s correlation coefficient. For categorical variables, we analyzed the differences in CEI and SSRI scores between the positive and negative groups of each category using a Student *t*-test.

All the statistical and machine learning analyses were conducted in Python 3.9.12.

## Results

### Demographics and clinical characteristics (Cohorts 1 and 2)

Demographics and clinical characteristics comparing the two Cohorts are in Table 1. The proportion of women in the CEI and SSRI groups was lower than that in Cohort 2. The proportion of Alzheimer's disease in the CEI group was higher than that in the SSRI group, whereas the proportion of mixed dementia in the CEI group was lower than that in the SSRI group. There were no significant differences in age, vascular risk factors, and other comorbidities among the Cohort 1 CEI group, Cohort 1 SSRI group, and Cohort 2.

### Volume differences between the benefitted and non-benefitted groups (Cohort 1)

**Fig. 1.**
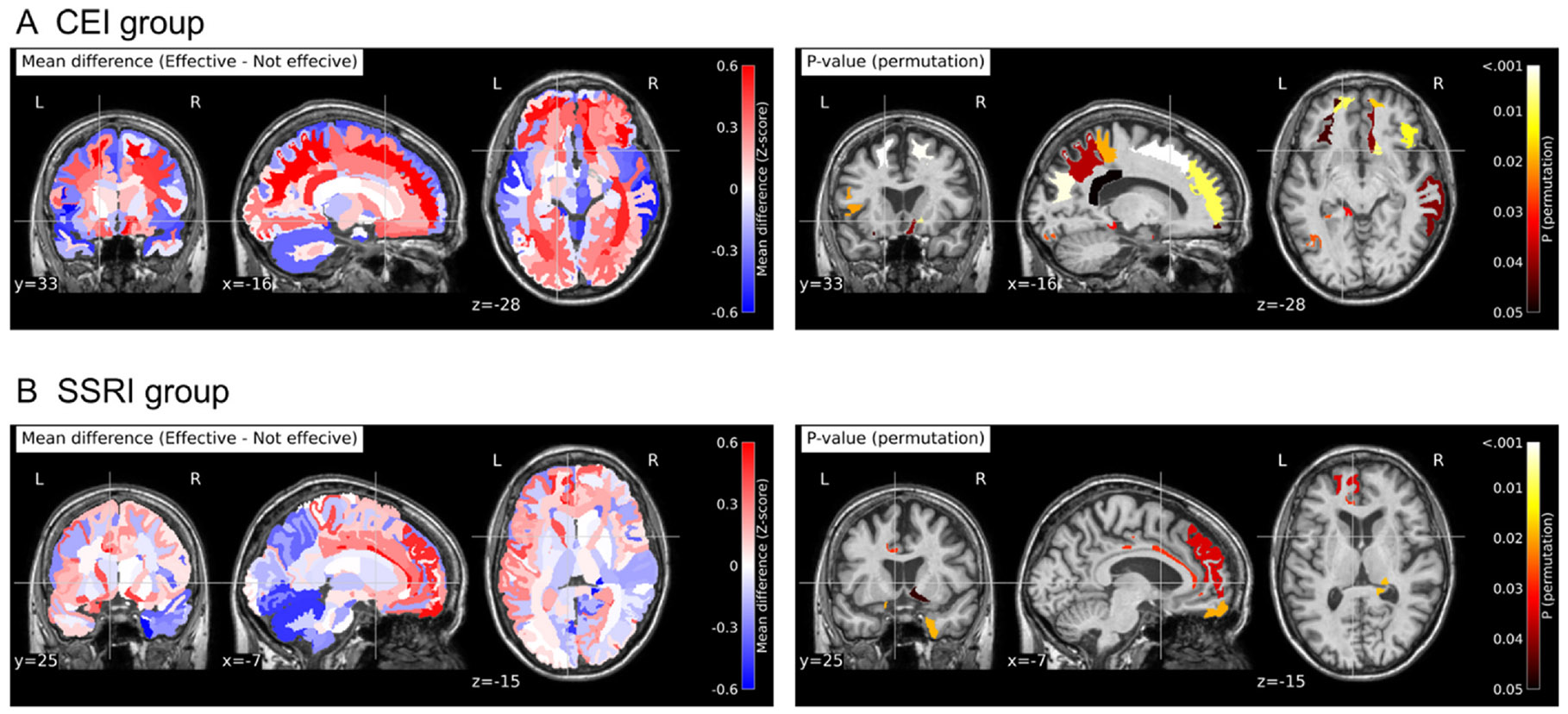
Heatmap visualization of the volume differences between the effective and non-effective individuals in the CEI (A) and SSRI (B) groups. In the left panel, each brain structure with the volume difference was color-coded, calculated by standardized volume of effective group minus standardized volume of non-effective group. In the right panel, each brain structure with the significant p-value was color-coded. CEI: cholinesterase inhibitors; L: left; R: right; SSRI: serotonin-specific reuptake inhibitors.

Significant differences in regional brain volumes were observed between the benefitted and non-benefitted groups for each medication type. Specifically, 22 anatomical brain structures showed differences between the CEI-benefitted and the CEI-non-benefitted groups. Similarly, nine anatomical structures exhibited differences between SSRI-benefitted and SSRI-non-benefitted groups, as detailed in Table S1. These regions included the anterior basal forebrain, encompassing the *nucleus basalis of Meynert* (nbM), where a greater volume correlated with treatment benefit for both CEI and SSRI. For visual representation, the results, including differences in z-score-transformed regional volumes and corresponding p-values, were superimposed on a representative T1-weighted image (Fig. 1).

### Performance and standardized beta coefficients of elastic net regression models (Cohort 1)

**Fig. 2.**
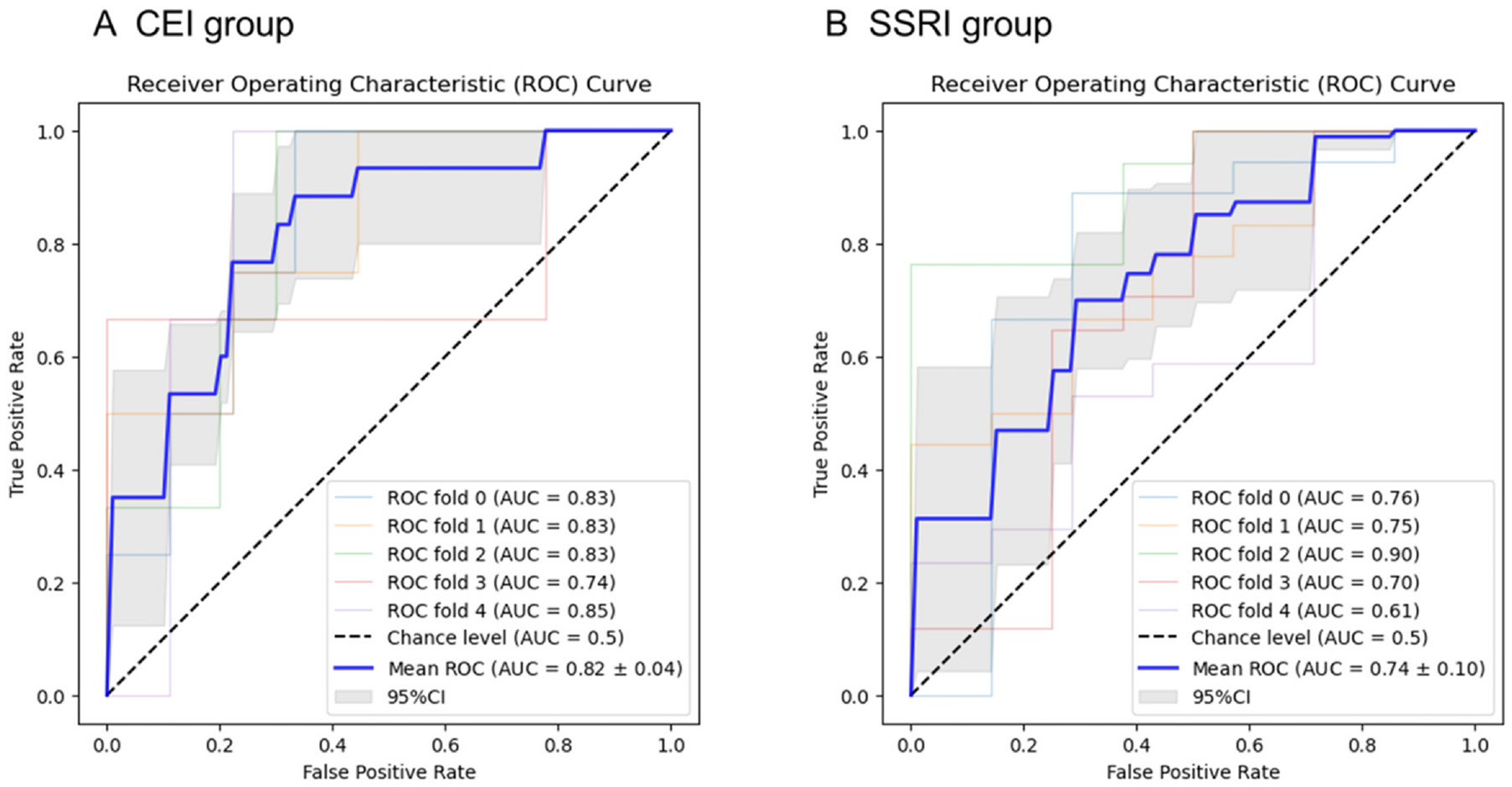
AUC-ROC from the stratified five cross-validation in the CEI (A) and SSRI (B) groups. The performance of each model achieved the AUC from the ROC analysis of 0.82 ± 0.04 for CEI and that of 0.74 ± 0.10 for SSRI. AUC: area under the curve; CEI: cholinesterase inhibitors; ROC: receiver operating characteristic curve; SSRI: serotonin-specific reuptake inhibitors.

The performance of each model was evaluated on Cohort 1 using AUC-ROC obtained from five-fold cross-validation. The results showed a significantly better classification than random. The AUC for predicting CEI effectiveness was 0.82 ± 0.04 (p < 0.001), while the AUC for predicting SSRI effectiveness was 0.74 ± 0.10 (p < 0.001), as illustrated in Fig. 2. The cut-off values, as determined by the Youden Index, were set at 0.33 for the CEI and 0.29 for the SSRI. The CEI score demonstrated a sensitivity of 0.88 and a specificity of 0.67, while the SSRI score showed a sensitivity of 0.70 and a specificity of 0.71. Table S2 summarizes the standardized beta coefficients of 22 anatomical structures in the CEI effectiveness prediction model and nine in the SSRI effectiveness prediction model. The anterior basal forebrain had the highest coefficient in the CEI model, whereas the nucleus accumbens had the highest in the SSRI model.

### Relationships between CEI score, SSRI score, demographic and clinical features (Cohort 2)

The CEI and SSRI prediction models provide scores representing the likelihood of a positive response: CEI score and SSRI score. The correlation between CEI and the SSRI scores in Cohort 2 was not significant (Pearson coefficient = 0.032, p = 0.254). Older age was associated with lower CEI and SSRI scores (Pearson coefficient of −0.19 and −0.18, p < 0.001 for both). Higher diastolic blood pressure corresponded with a greater CEI score (Pearson coefficient = 0.085, p = 0.0059), while lower systolic blood pressure and higher LDL levels were linked to greater SSRI scores (Pearson coefficient of −0.097 and 0.15, and p values of 0.0015 and 0.0015, respectively). Participants diagnosed with AD and VaD exhibited lower CEI and SSRI scores. Additionally, lower SSRI scores were noted in individuals with hypertension, diabetes, dyslipidemia, and peripheral vascular disease (Table 3).

## Discussion

We have developed models to predict the benefits of commonly prescribed medications based on a data-driven approach applied to clinical information obtained from EHR and brain MRI findings. Preserved volume in the anterior basal forebrain, including the Nucleus Basalis of Meynert (nbM), was associated with the benefits of both CEIs and SSRIs. The preservation of white matter volume in the medial-frontal, fronto-orbital, parietal, and occipital cortices was associated with CEI to benefit. In contrast, preservation of the Nucleus Accumbens and frontal cortices, such as the fronto-orbital cortex, anterior cingulate, and superior frontal gyrus, was associated with SSRI benefits. These neuroanatomical characteristics predicted CEI and SSRI scores, indicating treatment benefits.

The nbM is the origin of the cholinergic system. The preservation of this region was associated with CEI benefits, consistent with previous research. [29-31] Brain areas whose volume preservation was associated with CEI benefits were white matter areas where the Vesicular Acetylcholine Transporter (VAChT) is abundantly distributed. [42] VAChT is a neurotransmitter transporter located in presynaptic axons that loads acetylcholine into secretory vesicles, enabling its secretion at synapses. Thus, it can be inferred that, for CEIs to be beneficial, both the origin (nbM) and destination (VAChT) of cholinergic neurons must be preserved.

The *Nucleus Accumbens* (NAc) is linked to neuropsychiatric symptoms like depression and addiction [43,44], housing several serotonin receptors, including 5-HT2A and 5-HT_2 C_ [45]. Serotonergic transmission in the NAc regulates dopamine release, and impaired serotonin stimulation of dopamine is associated with anhedonia and lack of motivation, key factors in depression [44,46]. The nbM receives serotonergic input from the *Raphe* nuclei [47]. Brain areas whose volume preservation was associated with SSRI benefits include where the 5HT_2A_ receptor is abundantly distributed. [42,46,48] The 5-HT_2A_ receptor, a G protein-coupled receptor, is associated with psychiatric symptoms, including those seen in dementia such as depression, agitation, and hallucinations [49,50]. Therefore, we speculate that, for SSRIs to be effective, the volume of the NAc and areas receiving serotonergic input via 5-HT_2A_ receptors must be preserved.

Lower CEI and SSRI scores were related to older age, and diagnosis of AD and VaD. This may result from degeneration in cholinergic and serotoninergic systems linked to aging and AD and VaD pathologies, and may support the limited use of CEIs in early to mid-stage dementia and suggest less indication for advanced stages. Vascular risk factors such as elevated systolic blood pressure, peripheral vascular disease, hypertension, diabetes, and dyslipidemia were associated with lower SSRI scores. This suggested vulnerability of the serotonergic system to vascular pathologies. In contrast, higher diastolic blood pressure was linked to greater CEI scores, while elevated LDL levels correlated with higher SSRI scores. Further research is necessary to explore the relationships between vascular pathologies and responses to CEIs and SSRIs.

Our analysis of real-world clinical data from EHR offers insights into medication effects across diverse patient populations, enhancing the generalizability of findings beyond clinical trials [51,52]. Key challenges included data quality, biases from confounding factors, and variability in MRI scanners and parameters [53]. Real-world data sources often have inconsistent quality, leading to potential biases in analysis [54]. While clinical notes provide important information on treatment response, documentation varies, resulting in unclear descriptions of outcomes. To address this, we developed the NOTE score, which uses keywords for consistent evaluation [35]. For MRI data harmonization, we utilized a multi-atlas label fusion algorithm [36] and OPNested Combat methods [41]. Despite expected biases, the neuro-scientifically reasonable results support the robustness of our data harmonization approach.

The study had several limitations. First, inconsistencies in MRI field strength, vendor, scanning parameters, and scan timing made it difficult to eliminate biases in the predictive models. Second, while CEI (ROC-AUC of 0.82) and SSRI (ROC-AUC of 0.74) scores were promising indicators, they were not satisfactory for widespread clinical use. We anticipate that other factors influencing the benefits of these medications exist and warrant further investigation. Second, while the CEI (ROC-AUC of 0.82) and SSRI (ROC-AUC of 0.74) scores show promise, they are not satisfactory for widespread clinical use. We believe there are additional factors that can influence the effectiveness of these medications and require further investigation. These factors include drug-specific elements, such as the specific types of CEI or SSRI used, their dosage, and duration of treatment. Additionally, there are non-modifiable patient factors, such as age, sex, comorbidities, and genotypes related to drug sensitivity and risk of Alzheimer’s disease (e.g., ApoE genotype). Modifiable factors, including body composition, lifestyle, nutrition, cognitive reserve, and other medications that may interact with CEI or SSRI, also play a role. Since our study relied on retrospective observations, these factors were not controlled and often unavailable for analysis. Third, the reliance on manual EHR review limited the sample size for Cohort 1, affecting the model's ability to account for confounding variables. Future studies utilizing Natural Language Processing (NLP) to analyze EHRs will help overcome this limitation and expand the study cohort [55].

## Conclusions

We developed predictive models using real-world EHR and brain MRI scans from clinical practice to forecast responses to CEI and SSRI medications. The benefits of CEI or SSRI were linked to the volumes of anatomical structures associated with the cholinergic or serotonergic systems, respectively. While the responses to CEI and SSRI are independent of each other, a poor response to both medications correlated with old age and diagnosis of AD and VaD.

## Supplementary Material

supplementary material

## Figures and Tables

**Table 1:** Demographics and clinical characteristics.

	Cohort 1			
Variables	CEI (n = 58)	SSRI (n = 121)	Cohort 2 (n = 1244)	P-value
***Demographics***				
**Age, years**	72.1 ± 10.1	73.0 ± 8.9	71.1 ± 9.3	0.063
**Women**	26 (44.8)	56 (46.3)	695 (55.9)	0.012^a,b^
***Treatment Benefits***				
**Cognitive NOTE Score**	22 (37.9)	NA	NA	
**Psychiatric NOTE Score**	NA	100 (82.6)	NA	
***Vascular Risk Factors***				
**Systolic Blood Pressure, mmHg**	140.4 ± 20.2	141.5 ± 25.0	140.7 ± 24.5	0.439
**Diastolic Blood Pressure, mmHg**	74.8 ± 11.2	76.5 ± 14.3	76.9 ± 12.6	0.351
**Hypertension**	34 (58.6)	61 (50.4)	631 (50.7)	0.257
**Body Mass Index, kg/m** ^**2**^	26.5 (24.1 –;29.7)	25.7 (23.9 –;29.2)	26.6 (23.8 –;29.8)	0.713
**Obesity**	8 (13.8)	15 (12.4)	139 (11.2)	0.638
**Hemoglobin A1C, %**	5.9 (5.6 –;6.6)	5. (5.6 –;6.6)	6.0 (5.5 –;6.5)	0.812
**Diabetes**	13 (22.4)	23 (19.0)	264 (21.2)	0.534
**Low-Density Lipoprotein, mg/dl**	111.4 ± 37.5	108.2 ± 42.8	96.4 ± 41.2	0.066
**Dyslipidemia**	24 (41.3)	50 (41.3)	544 (43.7)	0.637
***Other Comorbidities***				
**Chronic Kidney Disease**	7 (12.1)	12 (9.9)	135 (10.9)	0.414
**Peripheral Vascular Disease**	2 (3.5)	5 (4.1)	89 (7.2)	0.192
**Heart Failure**	4 (6.9)	7 (5.8)	121 (9.7)	0.306
***Neuropsychiatric Diagnosis***				
**Alzheimer's Disease**	15 (25.9)	16 (13.2)	185 (14.9)	0.061
**Vascular Dementia**	4 (6.9)	24 (19.8)	89 (7.2)	< 0.001*,^b^
**Mixed Dementia**	7 (12.1)	14 (11.6)	84 (6.8)	0.058
**Others**	6 (10.3)	12 (9.9)	74 (5.1)	0.112

Data are mean ± standard deviation, number (%), or median (interquartile range).

P-value was derived from the Kruskal-Wallis or Fisher's exact tests based on the continuous or categorical variables of data among the CEI, SSRI, and Cohort 2 groups.

CEI: cholinesterase inhibitors; Mixed Dementia: Alzheimer's disease and vascular dementia; NA: not applicable; NOTE: note-based evaluation method for treatment efficacy; Others: frontotemporal dementia, Lewy body dementia, and other degenerative dementia; SSRI: serotonin-specific reuptake inhibitors.

* For the comparison between the CEI and SSRI groups (Bonferroni-corrected P < 0.05).

a For the comparison between the CEI group and Cohort 2 (Bonferroni-corrected P < 0.05).

b For the comparison between the SSRI group and Cohort 2 (Bonferroni-corrected P < 0.05).

**Table 2:** Field strength, vendors, and scan parameters from PMCoE database.

	Cohort 1		
MRI scanners	CEI (n = 58)	SSRI (n = 121)	Cohort 2 (n = 1244)
***Field Strength***			
**1.5-Tesla**	18 (31.0)	45 (37.2)	378 (30.4)
**3.0-Tesla**	40 (69.0)	76 (62.8)	866 (69.6)
***Vendors***			
**GE**	2 (3.4)	2 (1.6)	67 (5.4)
**Philips**	2 (3.4)	3 (2.5)	56 (4.5)
**Siemens**	54 (93.1)	116 (95.9)	1120 (90.0)
**Toshiba**	0 (0)	0 (0)	1 (0.0)
***Scan Parameters***			
**Resolution, mm** ^**3**^	1.0 (0.9 –1.2)	1.0 (0.8 –1.2)	1.0 (0.7 –1.2)
**Repetition Time, ms**	2110 (1200 –2300)	2110 (1500 –2300)	2110 (750 –2300)
**Echo Time, ms**	2.5 (1.9 –3.2)	3.0 (2.2 –3.9)	3.0 (1.9 –3.9)
**Inversion Time, ms**	900 (900 –1000)	900 (900 –1100)	900 (900 –976)

Data are number (%) or median (interquartile range). CEI: cholinesterase inhibitors; MRI: magnetic resonance imaging; PMCoE: Precision Medicine Center of Excellence for Alzheimer's Disease; SSRI: serotonin-specific reuptake inhibitors.

**Table 3:** Relationship between CEI/SSRI scores and clinical characteristics in participants from Cohort 2 (n = 1244).

	CEI score			SSRI score		
Continuous Variables	Pearson’s correlation coefficient		P-value	Pearson’s correlation coefficient		P-value
***Demographics***						
**Age, years**	−0.185		< 0.001	−0.177		< 0.001
***Vascular Risk Factors***						
**Systolic Blood Pressure, mmHg**	−0.022		0.481	−0.097		0.002
**Diastolic Blood Pressure, mmHg**	0.085		0.006	0.067		0.029
**Body Mass Index, kg/m** ^**2**^	0.043		0.161	0.03		0.332
**HbA1c, %**	−0.034		0.537	0.058		0.295
**LDL, mg/dl**	0.073		0.137	0.154		0.002
Categorical Variables	Positive Mean ± SD	Negative Mean ± SD	P–value	Positive Mean ± SD	Negative Mean ± SD	P–value
***Demographics (number among n = 1244)***						
**Women (n = 695)**	0.384 ± 0.243	0.396 ± 0.226	0.372	0.883 ± 0.207	0.868 ± 0.205	0.207
***Vascular Risk Factors***				
**Hypertension (n = 631)**	0.383 ± 0.239	0.395 ± 0.232	0.374	0.860 ± 0.215	0.893 ± 0.201	0.004
**Obesity (n = 139)**	0.385 ± 0.237	0.423 ± 0.235	0.078	0.876 ± 0.213	0.881 ± 0.194	0.766
**Diabetes (n = 264)**	0.375 ± 0.240	0.394 ± 0.234	0.245	0.834 ± 0.228	0.888 ± 0.198	< 0.001
**Dyslipidemia (n = 588)**	0.386 ± 0.223	0.393 ± 0.246	0.609	0.854 ± 0.206	0.896 ± 0.104	< 0.001
***Other Comorbidities***						
**Chronic Kidney Disease (n = 135)**	0.390 ± 0.253	0.385 ± 0.234	0.798	0.901 ± 0.213	0.873 ± 0.205	0.135
**Peripheral Vascular Disease (n = 121)**	0.417 ± 0.259	0.386 ± 0.233	0.170	0.821 ± 0.224	0.882 ± 0.203	0.002
**Heart Failure (n = 121)**	0.429 ± 0.253	0.385 ± 0.233	0.053	0.842 ± 0.234	0.880 ± 0.203	0.052
***Neuropsychiatric Diagnosis***						
**Alzheimer's Disease (n = 237)**	0.342 ± 0.208	0.401 ± 0.241	< 0.001	0.843 ± 0.197	0.884 ± 0.208	0.006
**Vascular Dementia (n = 96)**	0.367 ± 0.228	0.407 ± 0.241	0.003	0.853 ± 0.202	0.894 ± 0.202	< 0.001
**Mixed Dementia (n = 86)**	0.391 ± 0.234	0.389 ± 0.237	0.853	0.878 ± 0.214	0.872 ± 0.212	0.615
**Others (n = 82)**	0.392 ± 0.235	0.388 ± 0.236	0.805	0.870 ± 0.212	0.879 ± 0.203	0.485

The p-value of Pearson's correlation coefficient, when applied to continuous variables, tests the null hypothesis that the true population correlation is zero. Meanwhile, the p-value of the Student’s *t*-test, used for categorical variables, tests the null hypothesis that there are no differences in CEI and SSRI scores between the positive and negative groups within each category.

## Data Availability

Raw EHR data cannot be shared to protect patient information. Only fully de-identified data will be provided to researchers with institutional review board approval, qualification from the Johns Hopkins Richman Family Precision Medicine Center of Excellence, and a signed data use agreement with Johns Hopkins Medicine.

## Appendix A. Supporting information

Supplementary data associated with this article can be found in the online version at doi:10.1016/j.inpsyc.2025.100105.
