# Supplementary material for: Brain morphological characteristics predicting clinical response to selective serotonin reuptake inhibitors or cholinesterase inhibitors: A study of electronic medical records in patients with cognitive disorders
Source: Int Psychogeriatr. Author manuscript; Available in PMC 2026 Feb 12. (PMC12895311; doi:10.1016/j.inpsyc.2025.100105)
Supplement: supplementary material [file NIHMS2135389-supplement-supplementary_material.docx]

**Supplemental Material**

**Table S1. Anatomical Structures with Significant Differences In Volume Between the Benefitted and Non-benefitted Groups**

| CEI |  |  |
| --- | --- | --- |
| Anatomical structures | **Mean difference^1^** | **P-value** |
| Superior frontal white matter L | 0.898 | < 0.001 |
| Superior frontal white matter R | 0.788 | 0.002 |
| Superior occipital white matter L | 0.830 | 0.003 |
| Posterior corona radiata R | 0.782 | 0.005 |
| Anterior basal forebrain R | 0.709 | 0.007 |
| Superior prefrontal white matter L | 0.704 | 0.009 |
| Sylvian fissure parietal lobe part L | -0.674 | 0.011 |
| Inferior frontal white matter orbitralis R | 0.718 | 0.012 |
| Superior prefrontal white matter R | 0.547 | 0.017 |
| Postcentral white matter L | 0.630 | 0.020 |
| Inferior frontal gyrus triangularis L | -0.589 | 0.020 |
| Fusiform white matter L | 0.526 | 0.026 |
| Superior cerebellar peduncle R | -0.492 | 0.029 |
| Parahippocampal gyrus L | -0.492 | 0.032 |
| Precentral white matter R | 0.540 | 0.036 |
| Superior parietal white matter L | 0.599 | 0.038 |
| Rectus white matter R | 0.589 | 0.041 |
| Middle temporal gyrus R | -0.533 | 0.041 |
| Parietal lobe sulci R | -0.513 | 0.043 |
| Postcentral white matter R | 0.490 | 0.045 |
| Lateral front-orbital white matter L | 0.472 | 0.045 |
| Splenium of corpus callosum L | 0.492 | 0.049 |
| SSRI |  |  |
| Anatomical structures | **Mean difference^1^** | **P-value** |
| Anterior basal forebrain L | 0.611 | 0.012 |
| Fornix stria terminalis R | -0.559 | 0.018 |
| Fusiform white matter R | -0.582 | 0.019 |
| Middle front-orbital gyrus L | 0.555 | 0.019 |
| Cingulate gyrus L | 0.547 | 0.029 |
| Anterior basal forebrain R | 0.509 | 0.033 |
| Precuneus white matter R | -0.543 | 0.033 |
| Superior frontal gyrus L | 0.495 | 0.035 |
| Nucleus accumbens R | 0.447 | 0.047 |

^1^Standardized volume difference: volume of effective group minus volume of non-effective group. CEI: cholinesterase inhibitors; L: left; R: right; SSRI: serotonin-specific reuptake inhibitors.

**Table S2. Standardized Beta Coefficients of Anatomical Structures Selected by Elastic Net Regression Models**

| CEI |  |
| --- | --- |
| Anatomical structures | **Coefficient** |
| Anterior basal forebrain R | 0.0498 |
| Posterior corona radiata R | 0.0351 |
| Rectus white matter R | 0.0275 |
| Superior frontal white matter R | 0.0246 |
| Superior frontal white matter L | 0.0242 |
| Fusiform white matter L | 0.0230 |
| Superior occipital white matter L | 0.0217 |
| Inferior frontal white matter orbitralis R | 0.0207 |
| Splenium of corpus callosum L | 0.0173 |
| Postcentral white matter L | 0.0122 |
| Lateral front-orbital white matter L | 0.0108 |
| Superior prefrontal white matter L | 0.0092 |
| Superior prefrontal white matter R | 0.0091 |
| Postcentral white matter R | 0.0019 |
| Precentral white matter R | -0.0026 |
| Superior prefrontal white matter R | -0.0062 |
| Parietal lobe sulci R | -0.0164 |
| Sylvian fissure parietal lobe part L | -0.0288 |
| Middle temporal gyrus R | -0.0299 |
| Inferior frontal gyrus triangularis L | -0.0313 |
| Parahippocampal gyrus L | -0.0362 |
| Superior cerebellar peduncle R | -0.0397 |
| SSRI |  |
| Anatomical structures | **Coefficient** |
| Nucleus accumbens R | 0.0601 |
| Middle front-orbital gyrus L | 0.0479 |
| Cingulate gyrus L | 0.0428 |
| Superior frontal gyrus L | 0.0359 |
| Anterior basal forebrain R | 0.0149 |
| Anterior basal forebrain L | 0.0068 |
| Precuneus white matter R | -0.0748 |
| Fusiform white matter R | -0.0800 |
| Fornix stria terminalis R | -0.1055 |

CEI: cholinesterase inhibitors; L: left; R: right; SSRI: serotonin-specific reuptake inhibitors.
